# The “Sandwich” Schedule: A Well-Tolerated Adjuvant Treatment Both in Intermediate–High- and High-Risk Endometrial Cancer

**DOI:** 10.3390/curroncol29120722

**Published:** 2022-11-26

**Authors:** Annamaria Ferrero, Luca Fuso, Isabella Cipullo, Roberta Danese, Annalisa Rossi, Sergio Gribaudo, Daniela Attianese, Luca Pace, Saverio Danese, Nicoletta Biglia

**Affiliations:** 1 Academic Division Gynaecology and Obstetrics, Mauriziano Hospital, University of Torino, 10128 Torino, Italy; 2 Radiotherapy Division, Mauriziano Hospital, 10128 Torino, Italy; 3 Radiotherapy Division, AO Città della Salute e della Scienza, Sant’Anna Hospital, 10126 Torino, Italy; 4 Gynaecology and Obstetrics Division 4, AO Città della Salute e della Scienza, Sant’Anna Hospital, 10126 Torino, Italy

**Keywords:** endometrial cancer, chemotherapy, radiotherapy, sandwich schedule, toxicity

## Abstract

(1) Background: In intermediate–high- and high-risk endometrial cancer (EC), radiotherapy (RT) and chemotherapy (CT) play a basic role. However, there is controversy regarding the optimal timing of their combination. The “sandwich” schedule involves adjuvant CT followed by RT and subsequent CT. The aim of this study is to assess the tolerability and efficacy of the “sandwich” schedule. (2) Methods: A retrospective study was conducted in two gynecological oncology units in Torino, Italy, from 1 January 2003 until 31 December 2021. Intermediate–high- and high-risk patients with available clinical data were included. Compliance with treatment, CT and RT toxicities, disease-free survival (DFS), cancer-specific survival (CSS) and overall survival (OS) were analyzed. (3) Results: A total of 118 patients were selected: 27.1% FIGO I-II stages and 72.9% III-IV. Most of the patients (75.4%) received a carboplatin–paclitaxel combination, and as much as 94.9% of CT cycles were completed. Chemotherapy-related G3-4 toxicities were detected in 5.3% of the patients, almost half of which were hematological. Grade 2 gastrointestinal and genitourinary toxicities were reported in 8.4% and 4.2% of cases, respectively. With a median follow-up of 46 months, DFS was 77.6%, CSS was 70% and 5-year OS was 54%. (4) Conclusions: The “sandwich” schedule for CT and RT combination is an effective adjuvant treatment with low toxicity both in intermediate–high- and high-risk EC.

## 1. Introduction

Endometrial cancer (EC) is the fourth most common cause of cancer in women, with 10,013 new cases reported in Italy and 417,367 new cases reported worldwide in 2020 [1]. Most EC patients are diagnosed in an early stage (80% stage I), with a 5-year survival of more than 95%, which decreases if regional spread or distant metastases are detected (68% and 17%, respectively) [2,3,4].

The management of EC depends on several prognostic factors, especially FIGO stage, histological type, grading, myometrial invasion, lymphovascular invasion (LVSI) and lymph node metastasis [2,3,4]. The definition of prognostic categories has been updated from the 2015 ESMO-ESGO-ESTRO consensus conference to the 2020 ESGO/ESTRO/ESP guidelines including molecular classification [3,5]. Based on prognostic factors, the risk is summarized as low, intermediate, intermediate–high, high, advanced or metastatic according to the new guidelines for the management of EC [3,5].

The pivotal treatment for EC is surgery with cytoreductive intent, with mini-invasive procedures representing the best option, although in intermediate–high- and high-risk EC, radiotherapy (RT) and chemotherapy (CT) play a basic role [5]. Recent analyses demonstrate significant improvement in overall survival (OS) and disease-free survival (DFS) in association with CT and RT versus RT alone, especially in stage III disease [6,7]. 

The optimal timing of CT and RT is controversial. Few studies have compared the “sandwich” schedule, characterized by the alternation of CT–RT–CT, with the “sequential” schedule [6,8,9,10,11,12,13,14,15,16,17,18,19,20]. In a multi-institutional analysis published in 2019, Onal et al. reported a significantly long 5-year OS in patients treated with the “sandwich” schedule versus those treated with the “sequential” schedule (74% vs. 56%; *p* = 0.03) [20]. 

A challenge in adjuvant treatment is toxicity. Each type of adjuvant treatment has specific toxicities and, owing to potential serious side effects of drugs and radiation, it is difficult to design an improved schedule including both CT and RT. The administration of CT before RT can cause toxicity, blocking the subsequent administration of RT [16]. In contrast, performing RT before CT may lead to progression outside the radiation fields. Therefore, the “sandwich” schedule can be considered as a possible alternative [16,17,21,22,23]. 

The aim of this study is to assess tolerability and efficacy of the “sandwich” schedule for the adjuvant treatment of EC.

## 2. Materials and Methods

A retrospective study was conducted in two gynecological oncology units (Mauriziano and Sant’Anna Hospitals) in Torino, Italy. Patients with intermediate–high- and high risk EC with available clinical data and adequate follow-up were selected from 1 January 2003 to 31 December 2021. 

The following clinical data were collected from the computerized archives of the two divisions: age, type of surgery, histological type, grading, myometrial invasion, lympho-vascular space invasion (LVSI), staging according to FIGO 2009 and type of adjuvant treatments, focusing on their toxicities. 

CTACAE v. 4.0 was used to report the CT toxicity and the RTOG classification for the toxicity. 

Stage was classified according the FIGO 2009 staging, and patients staged according the FIGO 1988 criteria were reclassified to guarantee data uniformity. 

Compliance with treatment, CT and RT toxicities, disease-free survival (DFS) and overall survival (OS) were analyzed. Cox’s multivariate logistic regression was carried out to evaluate associations with OS and DFS. The results are expressed as a hazard ratio (HR) with a 95% confidence interval (95% CI); statistical significance was set at a p value of less than 0.05. Survival was assessed by Kaplan–Meier curves. The difference between the survival curves was assessed by log-rank test.

This study was approved by the Institutional Review Board and Ethics Committee A.O.U. Città della Salute e della Scienza di Torino/A.O. Ordine Mauriziano/ASL Città di Torino (protocol code 80/2022, 7 March 2022) and conducted in accordance with the principles of the Declaration of Helsinki. All patients signed informed consent to receive adjuvant treatment and have their clinical data included in the hospital databases for research analyses.

## 3. Results

### 3.1. Population

A total of 118 patients were identified. Patient characteristics are reported in Table 1. 

The median age of patients was 65 years. All patients included in the study underwent hysterectomy and a bilateral salpingo-oophorectomy. In many of the female (75 patients, 63.5%) lymphadenectomy was performed; 42 patients (35.6%) underwent pelvic lymphadenectomy, 21 (17.8%) underwent pelvic and lomboaortic lymphadenectomy and 8 (6.8%) underwent lymph node sampling, whereas more recently, 4 patients (3.3%) underwent sentinel node biopsy. A total of 19 patients (16.1%) underwent staging omentectomy. Peritoneal washing was performed in 87.2% of cases.

The predominant histology was endometrioid (55.9%), and non-endometrioid histology was detected in 44.1% of cases (15.3%, serous papillary; 8.5%, clear cell; 8.5%, Mullerian histotype or carcinosarcoma; 5.1%, undifferentiated; 3.4%, mixed). Grading was 3 in 66.6% of cases.

FIGO III-IV stages accounted for 72,9 % of cases; the most represented were IIIC1 with 35 cases (29.7%), and IIIA, with 26 cases (22%).

### 3.2. Adjuvant Treatments and Toxicities

#### 3.2.1. Chemotherapy

Almost all patients (98.3%) received platinum-based therapy (carboplatin or cisplatin). A proportion of 75.4% of patients (*n* = 89) received a carboplatin–paclitaxel combination, 22% (*n* = 26) received platinum plus anthracyclines (doxorubicin or epirubicin) and only 2.5% (*n* = 3) received other schedules (cisplatin as a single drug, epirubicin + cyclophosphamide, epirubicin + paclitaxel).

A proportion of 94.9% of prescribed cycles was completed. A dose reduction was necessary in three (2.5%) of the patients. The “sandwich” schedule mostly consisted of three cycles of platinum-based CT followed by external and/or brachy RT and then three more cycles of the same CT. 

Chemotherapy-related severe (G3-4) toxicities were detected in 5.3% of patients, almost half of which were hematological. Grade 2 gastrointestinal and genitourinary toxicities were reported in 8.4% and 4.2% of cases, respectively (Table 2).

#### 3.2.2. Radiotherapy

Radiation therapy was not performed in three patients, in two cases because of disease progression during the first cycles of chemotherapy and in the third case as a result of thrombotic complication. 

Treatment was administered according to the BOX technique in 64.4% of patients (*n* = 76), according to the VMAT technique in 31.3% if patients (*n* = 37) and according to the IMRT or tomotherapy technique in 2% of patients. The median dose delivered was 50.4 Gy (36–50.4 Gy). The median number of fractions was 28 (12–28). Brachytherapy was completed in 75 patients (63.5%). The median dose delivered was 10 Gy (5–20 Gy) in two fractions (1–4 fr).

Radiotherapy-related toxicities are reported in Table 3. Grade 1 gastrointestinal (GI) adverse events (i.e., mild nausea, an episode of vomiting within 24 h and slight increases in the frequency of daily evacuations) occurred in 23.7% of patients; 8.4% reported grade 2 GI toxicity (i.e., significant nausea, two to five vomiting episodes in 24 h, a significant increase in the number of day and night evacuations or constipation, the presence of ulcers and proctitis), and no patient referred grade 3 or grade 4 GI toxicity. Genitourinary (GU) toxicity was grade 1 in 16.1% of patients (microhematuria, mild proteinuria or sporadic dysuria) and grade 2 in 4.2% (macrohematuria without clot presence, medium-severity proteinuria or dysuria), and no patient experienced grade 3 or grade 4 GU toxicities. Most of the women included in this study did not develop any toxicities.

### 3.3. Recurrences

Recurrences occurred in 21.1% of patients (*n* = 25). 

Local recurrences occurred in 7.6% of patients (*n* = 9), mainly located in the peritoneum. Median disease-free interval was 18 months. 

Distant relapses occurred in 13.5% (*n* = 16) of patients and involved lymph nodes in 10.2% (*n* = 10); liver in 5.1% (*n* = 5); lungs in 4, 1% (*n* = 4); and pleura, brain and spine in fewer cases. The median time from the end of treatment until the onset of recurrence was 10.9 months. 

Only two patients developed both local and distant relapses.

### 3.4. Prognostic Factors for OS and DFS

Median follow-up (FU) time was 46 months.

At the end of the FU period, 51 patients (43.2%) were alive without disease, 10 patients (8.5%) were alive with disease, 40 patients (33.9%) died as a result of the disease (DOD—deceased of disease), 16 patients (13,6%) died from other causes (DOC—deceased for other causes) and one was lost to FU. DFS was 77.6%, CSS was 70% and 5-year OS 54%.

**Figure 1:**
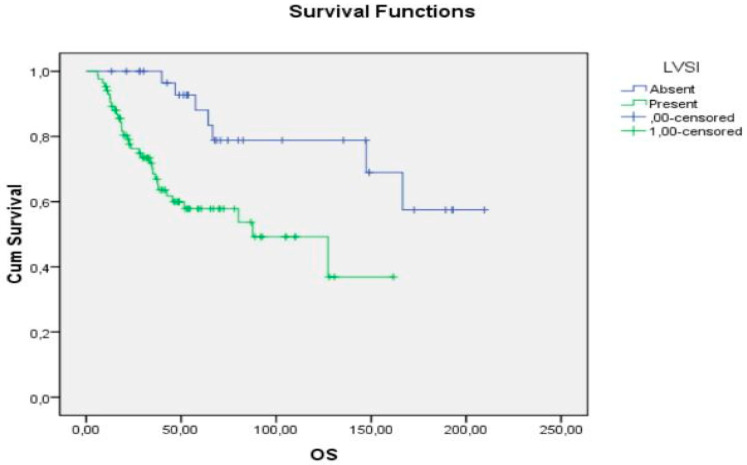
OS according to LVSI.

According to multivariate analysis of prognostic factors for OS, FIGO stage and LVSI were statistically significant. Actuarial OS was 60.5% for stages III-IV and 81.3% for stages I-II (*p* value, 0.002; OR, 2.8 (1.1–6.8)), whereas according to LVSI, OS was 78.8% if LVSI was absent and 61.2% at 5-year follow-up if LVSI was present (*p* value, 0.002; OR, 4.1 (1.6–10.4)) (Figure 1).

According to multivariate analysis of prognostic factors for DFS, only FIGO stage was found to be statistically significant (*p* value 0.06). DFS was 57% for stages III-IV at 5-year follow-up and 90% for stages I-II (*p* value, 0.009). 

## 4. Discussion

EC with unfavorable prognostic factors requires adjuvant treatment after surgery [3,5]. The combination of CT and RT seems to be the best approach for both local and remote disease control, but the optimal schedule combining CT and RT is controversial. Despite the recent publication of large, randomized trials (GOG 249, PORTEC3 and GOG 258) there is not yet a consensus on the optimal schedule and approach to treat intermediate–high- and high-risk EC, and the optimal timing of administration of adjuvant therapies and the number of CT cycles to be performed remain the subject of debate [24,25].

The “sandwich” schedule, which includes CT followed by RT and further CT, appears to be effective for the control of local and distant recurrences, combining the therapeutic effects of both methods.

There are few studies in the literature concerning the administration schedule of adjuvant treatments for EC, most of which report results with respect to advanced stages, excluding intermediate–high- and high-risk EC [16,17,21,22,23]. In contrast, our study includes both initial stages (27.6% FIGO I–II stages) and advanced stages (72.4% FIGO III-IV stages) at high risk of recurrence.

Compared with other studies, our population received a more homogeneous adjuvant therapy based on carboplatin and paclitaxel (75.4%), which is considered the current therapeutic standard. In addition, 98.3% of patients underwent platinum-based treatment. 

Concerning the chemotherapeutic toxicity of these treatments, our population experienced much less toxicity than that reported in other studies. Einstein et al. [8] and the PORTEC-3 trial [17] report 14% and 5–20% G3 hematological toxicity, respectively, compared with 5.9% in our series. RTOG 9708 [23] reports 10% overall hematological toxicity, whereas in our study, overall hematological toxicity was 8.3%. 

The radiotherapy toxicity detected in the PORTEC-3 trial [17] is significantly higher than that observed in our study, with G2 gastrointestinal toxicity of 44% vs. 7.6% reported in our study. Genitourinary toxicity (4.2% G2 toxicity) is also lower in our series compared to the 7% reported in the PORTEC-3 study [17] and 3% reported in the RTOG 9708 study [23]. These higher toxicities do not correspond to improved DFS or OS. 

Only Arden and colleagues (2020) analyzed the effects of adjuvant treatments (“sandwich” vs. non-“sandwich”) in a cohort of 107 patients, highlighting a significantly lower chronic overall toxicity for the “sandwich” patients (*p* 0.001) [26].

We can conclude that the global toxicity experienced by our population was generally lower than that reported in the literature, and patient compliance with therapy was favorable (94.9% of chemotherapy cycles completed).

Focusing on the prognostic outcomes, we found that the 5-year OS is correlated with FIGO stage and LVSI, with longer survival in patients with I–II stage and no LVSI, confirming that LVSI represents an independent prognostic factor [27].

In our study, actuarial OS was 60.5% for stages III-IV and 81.3% for stages I-II, in agreement with literature reports. Lupe et al. investigated a “sandwich” schedule comprising four cycles of adjuvant carboplatin and paclitaxel followed by pelvic RT, with two additional cycles of carboplatin and paclitaxel in a population of stage III and IVA EC and reported a 3-year OS of 68% [13]. In the GOG 258 trial, the population consisted of patients with endometrioid adenocarcinoma FIGO stage III/IV and residual tumor <2 cm or serous or clear-cell adenocarcinoma FIGO stage I–II, whereas the analyzed scheme was six cycles of carboplatin plus 175 mg/m^2^ paclitaxel versus percutaneous pelvic radiation with two cycles of 50 mg/m^2^ cisplatin followed by four cycles of carboplatin AUC-6 plus paclitaxel 175 mg/m^2^. In this trial, the OS after 5 years was 73% [28]. The PORTEC-3 trial reported significant improvement in 5-year OS in stage III EC patients treated with CT plus RT versus RT alone (78.5% versus 68.5%, respectively; HR 0.63; *p* = 0.043), whereas in stage I–II, 5-year OS was 83.8% with CT plus RT versus 82% with RT alone (HR 0.84; *p* = 0.50) [7]. In a recent multi-institutional analysis, Onal et al. demonstrated significantly longer 5-year OS rates in IIIC patients treated with the “sandwich” schedule than in the sequential arm (74% vs. 56%; *p* = 0.03) [20]. Verrengia and colleagues in Italy, studying a cohort of 36 patients with stage III EC, showed that optimal cytoreduction was a prognostic factor for OS and that adjuvant CT with RT seems to decrease the risk of recurrence but only with a borderline significance in OS [11]. In contrast, the GOG 258 trial, comparing CT+RT versus CT alone for advanced EC, did not report differences in DFS and OS, with more vaginal, pelvic and para-aortic relapses in group that received CT alone [9,28]. In 2020, Chen and colleagues investigated a series of 51 women with high-risk EC and found that a “sandwich” schedule was an independent predictor of longer OS (HR 0.07) compared with concurrent CT-RT [29]. In 2020, McEachron et al. compared CT-RT-CT (36.8% of the population) vs. CT followed by RT (28.9%) or RT followed by CT (34.2%) in a sample of 152 patients. CT-RT-CT demonstrated superiority over CT-CR and RT-CT sequencing in terms of 3-year PFS (55% vs. 34% and 37%, respectively) and 3-year OS (71% vs. 50% and 52%, respectively) [30].

An Italian experience was reported by Raspagliesi and colleagues in 2021, investigating outcomes and patterns of recurrence in a population of 45 women with III-IVA EC who had undergone “sandwich” adjuvant treatment. They detected relapses in 15 patients (9 at a single site and 6 in multiple sites), concluding that the “sandwich” regimen is safe and associated with a reduced risk of locoregional recurrences, although the distant recurrence rate was increased [31].

In 2021, Hathout et al. investigated a sample of 686 patients, of whom 42.5% underwent sequential CT-RT, 25% underwent a sandwich schedule and 16.5 % underwent concurrent CT-RT; a 5-year OS of 74% was calculated, and the sequence and type of adjuvant therapy did not correlate with OS [32]. 

In 2022, Wang et al. published a retrospective study on 66 patients who underwent complete surgical staging followed by sandwich chemoradiotherapy (39 patients) or CT alone (27 patients). They found that the sandwich schedule was a positive predictor of 5-year DSS (*p* = 0.029), even if it was associated with higher acute hematologic toxicity than the CT-alone arm [33].

In our study, the 5-years DFS, depending on the FIGO stage, was 57% for stages III-IV and 90% for stages I-II (*p* value 0.009). These data seem to be in line with those reported in the literature. The PORTEC-3 trial reported a significant improvement in 5-year failure-free survival in stage III EC treated with CT plus RT versus RT alone (70.9% versus 58.4%, respectively (HR 0.61; *p* = 0·011)), whereas in stage I-II, it was 81.3% with chemoradiotherapy versus 77.3% with radiotherapy alone (HR 0.87; *p* = 0.54) [7]. In the multi-institutional analysis by Onal, 5-year DFS was 65% in patients who had undergone “sandwich” schedule, versus 54% in the sequential arms (*p* = 0.05) [20]. In the series reported by Glasgow et al. (41 patients with stage IIIA and IIIC EC) the 4-year DFS was 69% [12]. 

A summary of available evidence compared with the data obtained in the current study is reported in Table 4.

Our results show a lower treatments toxicity and superimposable OS and DFS compared to the studies reported in literature.

A limitation of this study is the retrospective design, not directly comparing “sandwich” and sequential schemes. Few non-randomized studies have compared the schemes, and there is considerable variability in terms of patient selection concerning FIGO stages, histotypes and therapeutic schedules. Our study presents a broader spectrum of stages, especially including those of a lower degree, as well as intermediate–high- or high-risk cases, which are rarely investigated in other series.

Another limitation is the long period of study, although the incidence of surgical treatment and adjuvant medical therapy was equal over time. Moreover, the stage of the older cases was updated according to the recent FIGO classification. Few patients underwent sentinel node biopsy, as it has been introduced as routine procedure in our institutions only this year. The identification of a sentinel lymph node is extremely important in EC, especially for early-stage disease, to define the risk of recurrence and plan an adequate adjuvant treatment avoiding more invasive surgical procedures.

In the era of molecular classification of EC, as well as on the basis of our results, we stress the importance of the preoperative mapping through imaging, as well as histological and molecular analyses of hysteroscopic biopsies to predict the low or high risk for nodal involvement upon preoperative evaluation [34]. 

Additional studies are necessary to confirm our data, but the “sandwich” schedule remains a suitable option when CT and RT are indicated in the adjuvant treatment.

## 5. Conclusions

Endometrial cancer includes several histological types and various stages under a single name, with varying prognoses. Therefore, it is complex to identify a unique therapeutic line, even following the analysis of several studies comparing different adjuvant therapies. The optimal timing of adjuvant CT and RT is controversial, as few studies have compared the “sequential” with the “sandwich” schedule. The best administration scheme for these two therapeutic methods remains the subject of debate.

Our study shows that a population with intermediate–high or high risk EC treated with the “sandwich” schedule experienced a very low rate of toxicity, whereas OS and DFS rates were in line with those reported in the literature. The “sandwich” schedule is a valid option, based on the high compliance with treatment, as well as in intermediate–high-risk patients, when CT can be considered.

## Figures and Tables

**Table 1:** Population.

Characteristic	Number (118 Patients)	Percentage (Range)
Age (median)	65	(36–83)
FIGO stage		
IA	5	4.2%
IB	14	11.9%
II	13	11.0%
IIIA	26	22.0%
IIIB	7	5.9%
IIIC1	35	29.7%
IIIC2	8	6.8%
IVA	3	2.5%
IVB	7	5.9%
FIGO I-II	32	27.1%
FIGO III-IV	86	72.9%
Histotype		
Endometrioid	66	55.9%
Non-endometrioid	52	44.1%
Grade		
1	4	2.5%
2	34	28.8%
3	81	66.6%
Myometrial invasion		
<50%	21	17.8%
>50%	97	82.2%
LVSI		
Present	85	72.0%
Absent	15	12.7%
Not known	18	15.3%

**Table 2:** Chemotherapy-related toxicities.

Chemotherapy-Related Toxicity	Number (118 Patients)	Percentage (Range)
**Neurologic**		
G1	5	4.2%
G2	3	2.5%
G3	0	0
**G3 hematological toxicity**		
Platelets	0	0
Neutrophiles	5	4.2%
Haemoglobin	2	1.7%
**G4 hematological toxicity**		
Platelets	0	0
Neutrophiles	2	1.6%
Haemoglobin	1	0.8%
**Thrombosis**	3	2.5%
**Allergic reaction to paclitaxel**	6	5.0%

**Table 3:** Radiotherapy-related toxicities.

Radiotherapy-Related Toxicities	Number (118 Patients)	Percentage (Range)
**Genitourinary**		
G1	19	16.1%
G2	5	4.2%
G3	0	0
G4	0	0
**Gastrointestinal**		
G1	28	23.7%
G2	9	7.6%
G3	0	0
G4	0	0

**Table 4:** Summary of available evidence.

	RTOG 9708 [23]	LUPE et al. [13]	EINSTEIN et al. [8]	GLASGOW et al. [12]	PORTEC-3 [7,17]	ONAL et al. [20]	HATHOUT et al. [32]	WANG et al. [33]	Current Study
**FIGO stage**	IB-IIIC	III-IV	I-IV	IIIA, IIIC	I-III	IIIC	IIIC	III	I-IV
**Schedule**	RT(+CDDP)+4CT	4CT +RT +2CT	3CT +RT +3CT	3CT +RT +3CT	RT(+CDDP) +4CT vs. RT	6CT+RT vs. 3CT+RT+ 3CT	6CT+RT (42.5%) vs. 3CT+RT+3CT (25%) vs. RT(+CDDP)+CT	3CT+RT +3CT vs. CT alone	3CT +RT +3CT
**OS**	77% (4 y)	68% (3 y)	84% (stages I-II, 3 y) 50% (stages III-IV, 3 y)	70% (5 y)	84% (stages I-II, 5 y) vs. 82% 78.5% (stages III-IV, 5 y) vs. 68.5%	56% (5 y) vs. 74% (5 y)	74% (5 y)	87% (5 y) vs. 77% (5 y)	81.3% (stages I-II, 5 y) 60.5% (stages III-IV, 5 y)
**CT-related toxicities**	H G3 1% H G4 0%	-	H G3 14% H G4 12.9%	-	H G3 5–20%	H G3 12% vs. 9%	-	H G3-G4 59% vs. 25%	H G3 5.9% H G4 2.4%
**RT-related toxicities**	GU G1 5% GU G2 3%	-	-	-	GI G2 44% GI G3-G4 14% GU G2 7%	GI G1-G2 56% vs. 54% GI G3-G4 2% vs. 2.1% GU G1-G2 40% vs. 36%	-	-	GU G1 16.1% GU G2 4.2% GI G1 23.7% GI G2 7.6%

CT = Chemotherapy; RT = Radiotherapy; OS = Overall survival; H = Hematological; GU = Genito-urinary; GI = Gastro-intestinal; y = years.

## Data Availability

Data are collected in the institutional database.

## References

[1] World Health Organization (2018). GLOBOCAN 2018: Estimated Cancer Incidence, Mortality and Prevalence Worldwide in 2018. http://gco.iarc.fr/today/data/factsheets/cancers/24-Corpus-uteri-fact-sheet.pdf.

[2] Associazione Italiana Oncologia Medica (2019). Linee Guida AIOM Neoplasie Dell’utero: Endometrio e Cervice. https://www.aiom.it/wp-content/uploads/2019/10/2019_LG_AIOM_Utero.pdf.

[3] Colombo N., Creutzberg C., Amant F., Bosse T., González-Martín A., Ledermann J., Marth C., Nout R., Querleu D., Mirza M.R. (2016). ESMO-ESGO-ESTRO Endometrial Consensus Conference Working Group. ESMO-ESGO-ESTRO Consensus Conference on Endometrial Cancer: Diagnosis, treatment and follow-up. Ann. Oncol..

[4] Burke W.M., Orr J., Leitao M., Salom E., Gehrig P., Olawaiye A.B., Brewer M., Boruta D., Villella J., SGO Clinical Practice Endometrial Cancer Working Group (2014). Society of Gynecologic Oncology Clinical Practice Committee. Endometrial cancer: A review and current management strategies: Part I. Gynecol. Oncol..

[5] Concin N., Matias-Guiu X., Vergote I., Cibula D., Mirza M.R., Marnitz S., Ledermann J., Bosse T., Chargari C., Fagotti A. (2021). ESGO/ESTRO/ESP guidelines for the management of patients with endometrial carcinoma. ESGO/ESTRO/ESP guidelines for the management of patients with endometrial carcinoma. Int. J. Gynecol. Cancer.

[6] Gómez-Raposo C., Merino Salvador M., Aguayo Zamora C., Casado Saenz E. (2020). Adjuvant chemotherapy in endometrial cancer. Cancer Chemother. Pharmacol..

[7] De Boer S.M., Powell M.E., Mileshkin L., Katsaros D., Bessette P., Haie-Meder C., Ottevanger P.B., Ledermann J.A., Khaw P., D’Amico R. (2019). Adjuvant chemoradiotherapy versus radiotherapy alone in women with high-risk endometrial cancer (PORTEC-3): Patterns of recurrence and post-hoc survival analysis of a randomised phase 3 trial. Lancet Oncol..

[8] Einstein M.H., Frimer M., Kuo D.Y., Reimers L.L., Mehta K., Mutyala S., Huang G.S., Hou J.Y., Goldberg G.L. (2012). Phase II trial of adjuvant pelvic radiation “sandwiched” between combination paclitaxel and carboplatin in women with uterine papillary serous carcinoma. Gynecol Oncol..

[9] Boothe V., Orton A., Kim J., Poppe M.M., Werner T.L., Gaffney D.K. (2019). Does early chemotherapy improve survival in advanced endometrial cancer?. Am. J. Clin. Oncol. Cancer Clin. Trials.

[10] Lan C., Huang X., Cao X., Huang H., Feng Y., Huang Y., Liu J. (2013). Adjuvant docetaxel and carboplatin chemotherapy administered alone or with radiotherapy in a “sandwich” protocol in patients with advanced endometrial cancer: A single-institution experience. Expert Opin. Pharmacother..

[11] Verrengia A., Sigismondi C., Iannacone E., Bellia A., Busci L., Trezzi G., Malandrino C., Gianatti A., Frigerio L. (2020). Does cytoreductive surgery followed by adjuvant chemo-radiotherapy decrease the risk of recurrence and death in stage III endometrial cancer?. Tumori J..

[12] Gasgow M., Vogel R.I., Burgart J., Argenta P., Dusenbery K., Geller M.A. (2016). Long term follow-up of a phase II trial of multimodal therapy given in a ‘sandwich’ method for stage III, IV, and recurrent endometrial cancer. Gynecol. Oncol. Res. Pract..

[13] Lupe K., D’Souza D.P., Kwon J.S., Radwan J.S., Harle I.A., Hammond J.A., Carey M.S. (2009). Adjuvant carboplatin and paclitaxel chemotherapy interposed with involved field radiation for advanced endometrial cancer. Gynecol. Oncol..

[14] Geller M.A., Ivy J.J., Ghebre R., Downs L.S., Judson P.L., Carson L.F., Jonson A.L., Dusenbery K., Vogel R.I., Boente M.P. (2011). A phase II trial of carboplatin and docetaxel followed by radiotherapy given in a “Sandwich” method for stage III, IV, and recurrent endometrial cancer. Gynecol. Oncol..

[15] Geller M.A., Ivy J., Dusenbery K.E., Ghebre R., Isaksson Vogel R., Argenta P.A. (2010). A single institution experience using sequential multi-modality adjuvant chemotherapy and radiation in the ‘sandwich’ method for high risk endometrial carcinoma. Gynecol. Oncol..

[16] Dogan N.U., Yavas G., Yavas C., Ata O., Yilmaz S.A., Celik C. (2013). Comparison of ‘sandwich chemo-radiotherapy’ and six cycles of chemotherapy followed by adjuvant radiotherapy in patients with stage IIIC endometrial cancer: A single center experience. Arch. Gynecol. Obstet..

[17] De Boer S.M., Powell M.E., Mileshkin L., Katsaros D., Bessette P., Haie-Meder C., Ottevanger P.B., Ledermann J.A., Khaw P., Colombo A. (2016). Toxicity and quality of life after adjuvant chemoradiotherapy versus radiotherapy alone for women with high-risk endometrial cancer (PORTEC-3): An open-label, multicentre, randomised, phase 3 trial. Lancet Oncol..

[18] Secord A.A., Havrilesky L.J., O’Malley D.M., Bae-Jump V., Fleming N.D., Broadwater G., Cohn D.E., Gehrig P.A. (2009). A multicenter evaluation of sequential multimodality therapy and clinical outcome for the treatment of advanced endometrial cancer. Gynecol. Oncol..

[19] Bie Y., Zhang Z., Wang X. (2015). Adjuvant chemo-radiotherapy in the ‘sandwich’ method for high risk endometrial cancer-a review of literature. BMC Womens Health.

[20] Onal C., Sari S.Y., Yildirim B.A., Yavas G., Gultekin M., Guler O.C., Akyurek S., Yildiz F. (2019). A multi-institutional analysis of sequential versus ‘sandwich’ adjuvant chemotherapy and radiotherapy for stage IIIC endometrial carcinoma. J. Gynecol. Oncol..

[21] Zakem S.J., Robin T.P., Smith D.E., Amini A., Stokes W.A., Lefkowits C., Fisher C.M. (2019). Evolving trends in the management of high-intermediate risk endometrial cancer in the United States. Gynecol. Oncol..

[22] Chodavadia P.A., Jacobs C.D., Wang F., Havrilesky L.J., Chino J.P., Suneja G. (2020). Off-study utilization of experimental therapies: Analysis of GOG249-eligible cohorts using real world data. Gynecol. Oncol..

[23] Greven K., Winter K., Underhill K., Fontenesci J., Cooper J., Burke T. (2006). Final analysis of RTOG 9708: Adjuvant postoperative irradiation combined with cisplatin/paclitaxel chemotherapy following surgery for patients with high-risk endometrial cancer. Gynecol. Oncol..

[24] Hamilton C.A., Pothuri B., Arend R.C., Backes F.J., Gehrig P.A., Soliman P.T., Thompson J.S., Urban R.R., Burke W.M. (2021). Endometrial cancer: A society of gynecologic oncology evidence-based review and recommendations. Gynecol. Oncol..

[25] Van Den Heerik A.S.V.M., Horeweg N., De Boer S.M., Bosse T., Creutzberg C.L. (2021). Adjuvant therapy for endometrial cancer in the era of molecular classification: Radiotherapy, chemoradiation and novel targets for therapy. Int. J. Gynecol. Cancer.

[26] Arden J.D., Marvin K., Nandalur S.R., Al-Wahab Z., Field J., Gadzinski J., Rakowski J.A., Rosen B., Jawad M.S. (2020). Sequencing of Adjuvant Chemoradiation for Advanced Stage Endometrial Cancer: Outcomes and Toxicity Profiles. Am. J. Clin. Oncol..

[27] Tortorella L., Restaino S., Zannoni G.F., Vizzielli G., Chiantera V., Cappuccio S., Gioè A., La Fera E., Dinoi G., Angelico G. (2021). Substantial lymph-vascular space invasion (LVSI) as predictor of distant relapse and poor prognosis in low-risk early-stage endometrial cancer. J. Gynecol. Oncol..

[28] Emons G., Tempfer C., Battista M.J., Mustea A., Vordermark D. (2018). Statement of the Uterus Committee of the Gynaecological Oncology Working Group (AGO) on the PORTEC-3 study. Geburtshilfe Frauenheilkd.

[29] Chen H.H., Ting W.H., Sun H.D., Wei M.C., Lin H.H., Hsiao S.M. (2020). Predictors of survival in women with high-risk endometrial cancer and comparisons of sandwich versus concurrent adjuvant chemotherapy and radiotherapy. Int. J. Environ. Res. Public Health.

[30] McEachron J., Zhou N., Spencer C., Shanahan L., Chatterton C., Singhal P., Lee Y.C. (2020). Evaluation of the optimal sequence of adjuvant chemotherapy and radiation therapy in the treatment of advanced endometrial cancer. J. Gynecol. Oncol..

[31] Raspagliesi F., Bogani G., Pinelli C., Casarin J., Cerrotta A.M., Delle Curti C.T., Ditto A., Chiappa V., Bosio S., Bertolina F. (2021). Patterns of failure after adjuvant “sandwich” chemo-radio-chemotherapy in locally advanced (stage III-IVA) endometrial cancer. J. Cancer Res. Clin. Oncol..

[32] Hathout L., Wang Y., Wang Q., Vergalasova I., Elshaikh M.A., Dimitrova I., Damast S., Li J.Y., Fields E.C., Beriwal S. (2021). A Multi-Institutional Analysis of Adjuvant Chemotherapy and Radiation Sequence in Women With Stage IIIC Endometrial Cancer. Int. J. Radiat. Oncol. Biol. Phys..

[33] Wang S.J., Wang L., Sun L., Shih Y.H., Hsu S.T., Liu C.K., Hwang S.F., Lu C.H. (2022). Outcomes of “sandwich” chemoradiotherapy compared with chemotherapy alone for the adjuvant treatment of FIGO stage III endometrial cancer. Front. Oncol..

[34] Cignini P., Vitale S.G., Laganà A.S., Biondi A., La Rosa V.L., Cutillo G. (2017). Preoperative work-up for definition of lymph node risk involvement in early stage endometrial cancer: 5-year follow-up. Updates Surg..

